# Geographical distribution of salmonella infected pig, cattle and sheep herds in Sweden 1993-2010

**DOI:** 10.1186/1751-0147-53-51

**Published:** 2011-10-05

**Authors:** Susanna Sternberg Lewerin, Lars Skog, Jenny Frössling, Helene Wahlström

**Affiliations:** 1 National Veterinary Institute, Department of Disease Control and Epidemiology, SE-751 89 Uppsala, Sweden; 2 Swedish University of Agricultural Sciences, Department of Biomedical Sciences and Veterinary Public Health, Box 7028, SE-750 07 Uppsala, Sweden; 3 Royal Institute of Technology, Geoinformatics, SE-100 44 Stockholm, Sweden; 4 Swedish University of Agricultural Sciences, Department of Animal Environment and Health, Box 7084, SE-750 07 Uppsala, Sweden

## Abstract

**Background:**

The Swedish salmonella control programme covers the entire production chain, from feed to food. All salmonella serotypes are notifiable. On average, less than 20 cases of salmonella in food-producing animals are reported every year. In some situations, the cases would be expected to cluster geographically. The aim of this study was to illustrate the geographic distribution of the salmonella cases detected in pigs, cattle and sheep.

**Methods:**

Data on all herds with pigs, cattle and sheep found to be infected with salmonella during the time period from 1993 to 2010 were obtained from the Swedish Board of Agriculture. Using the ArcGIS software, various maps were produced of infected herds, stratified on animal species as well as salmonella serotype. Based on ocular inspection of all maps, some were collapsed and some used separately. Data were also examined for temporal trends.

**Results:**

No geographical clustering was observed for ovine or porcine cases. Cattle herds infected with Salmonella Dublin were mainly located in the southeast region and cattle herds infected with Salmonella Typhimurium in the most southern part of the country. Some seasonal variation was seen in cattle, but available data was not sufficient for further analyses.

**Conclusions:**

Analyses of data on salmonella infected herds revealed some spatial and temporal patterns for salmonella in cattle. However, despite using 18 years' of data, the number of infected herds was too low for any useful statistical analyses.

## Background

The Swedish National salmonella control programme covers the entire production chain, from feed to food [1]. The salmonella monitoring and control are governed by the Law on Zoonoses [2] and several regulations [3,4]. All serotypes of salmonella are regarded as equally unacceptable and the legislation includes all serotypes and all animal species. All findings of *Salmonella *spp. in feed, animals or food of animal origin are notifiable and action is always taken to eliminate the infection. Whenever salmonella is isolated, restrictions on animal movements and manure are immediately put on the farm and a prompt investigation and trace-back of the infection is performed. An eradication plan is instituted by an official veterinarian and approved by the Swedish Board of Agriculture. The herd is not declared free from infection until all animals in the herd are negative in two consecutive faecal samplings one month apart, and adequate cleaning and disinfection have been completed. Culture is the detection method of choice for most samples from animals, as this is independent of serotype and has a high specificity, both important features for the Swedish situation. However, culture from faecal material has a low sensitivity [5] and may only be reliably used on herd level. This fact is accounted for in the control strategies.

Regular sampling of pig and cattle carcasses is performed at slaughterhouses. Furthermore, random lymph node samples are taken at slaughter. No regular sampling is performed in herds with pigs or ruminants, but a voluntary surveillance programme is in place for breeding pigs. Due to the pyramidal structure of the commercial pig production, about 60-65% of all slaughtered pigs descend directly from these breeding herds. Clinical or post-mortem suspicion of salmonella infection is notifiable for all veterinarians, who must take relevant samples in case of such suspicions. Salmonella samples are also taken at most post-mortem investigations of cattle and pigs.

Only a few cases of salmonella in food-producing animals are reported every year [6]. In some situations, the cases would be expected to cluster geographically. The aim of this study was to illustrate the geographic distribution of the salmonella cases detected in pigs, cattle and sheep.

## Methods

Data on all herds with pigs, cattle and sheep found to be infected with salmonella during the time period from 1993 to 2010 were obtained from the Swedish Board of Agriculture (SJV).

A herd was considered a case if salmonella was isolated from at least one faecal sample in the herd. Each holding was only included once even if investigations revealed that several animal species were infected. The case species was set to the animal species from which Salmonella was first identified, and thus regarded as the species of the herd. In the studied period there were a total of 267 holdings where salmonella was isolated from the animals. One large feed-borne outbreak involving 31infected pig herds occurred during the study period and only the first case of this outbreak was kept in the dataset (i.e. 30 of these herds were excluded) [7]. The final dataset included 13 sheep flocks, 200 cattle herds and 54 pig herds.

Analysis of the data was done for all *Salmonella *spp. as well as separately for the most common serotypes. Furthermore serotypes were grouped into cattle-, pig- and sheep-adapted types (*S*. Dublin, *S*. Derby and *S. diarizonae*) and "other" representing mainly feed-associated serotypes, and summarised by animal species. Finally, analyses were also done for serotypes associated with small passerine birds (*S*. Typhimurium DT U277 and DT40) [8,9].

Because the exact coordinates of each farm from the earlier years were not available, and such detailed resolution was not deemed necessary, postal codes were used for geographical reference. A few of the postal codes had been changed since the data was first collected. Based on the address or if that was not possible, the municipality, new postal codes were obtained.

Using various techniques in the software ArcGIS 10 (ESRI; Redlands, CA, USA), each case has been attributed with geographical coordinates representing the corresponding geometric centres, centroids, of the 5 digit postal code areas. Information for all cases has been stored in a geodatabase. From this geodatabase separate animated map series were generated for all serotypes or groups of serotypes isolated from pigs, cattle and sheep respectively. The maps were generated for each year and for each salmonella serotype, and subsequently amalgamated into versions conveying relevant information.

Ocular inspection of the maps was made before deciding what maps would be used separately and what maps could be collapsed into one.

In order to relate the distribution of cases to the geographical distribution of the animal populations, maps illustrating the animal density per 3 digit postal code area were created. For cattle, this was based on the average number of animals > 1 year of age on each holding in Sweden in 2008, as registered in the national database of individual cattle (Swedish Board of Agriculture). For pigs, the number of animals on each holding was based on data retrieved from the Swedish Board of Agriculture and the Swedish Animal Health Service in a previous study [10].

## Results

**Figure 1:**
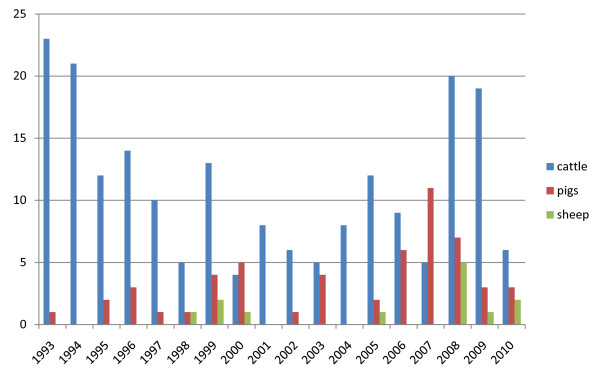
**Number of salmonella infected herds of different animal species in Sweden 1993-2010**.

**Figure 2:**
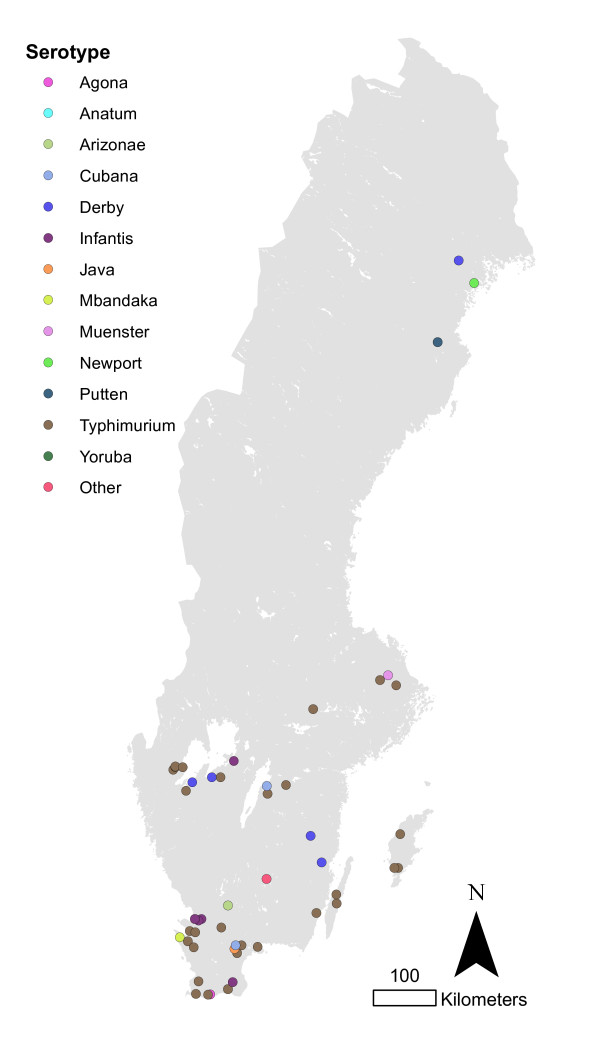
**Salmonella infected pig herds in Sweden 1993-2010**.

**Figure 3:**
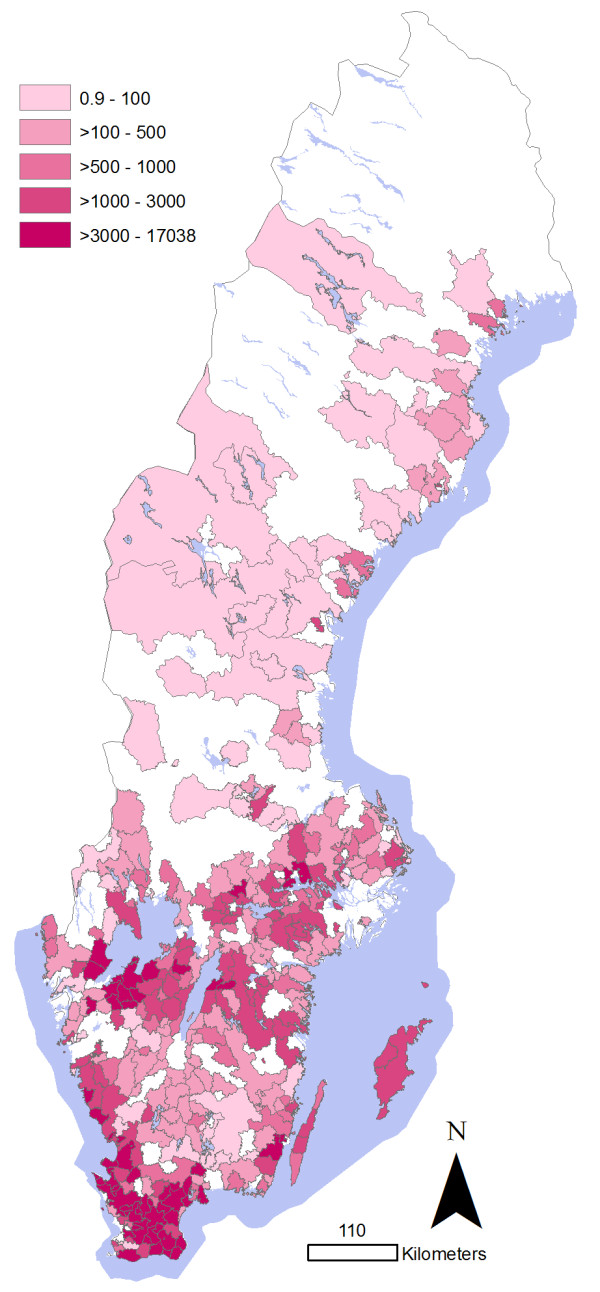
**Density of pigs by 3 digit postal code area in Sweden in 2007**. Density represents the number of animals, including adults, weaners, growers and fattening pigs, per 100 km^2^. ^© ^Lantmäteriet Gävle 2009, permission number I 2009/0830.

**Figure 4:**
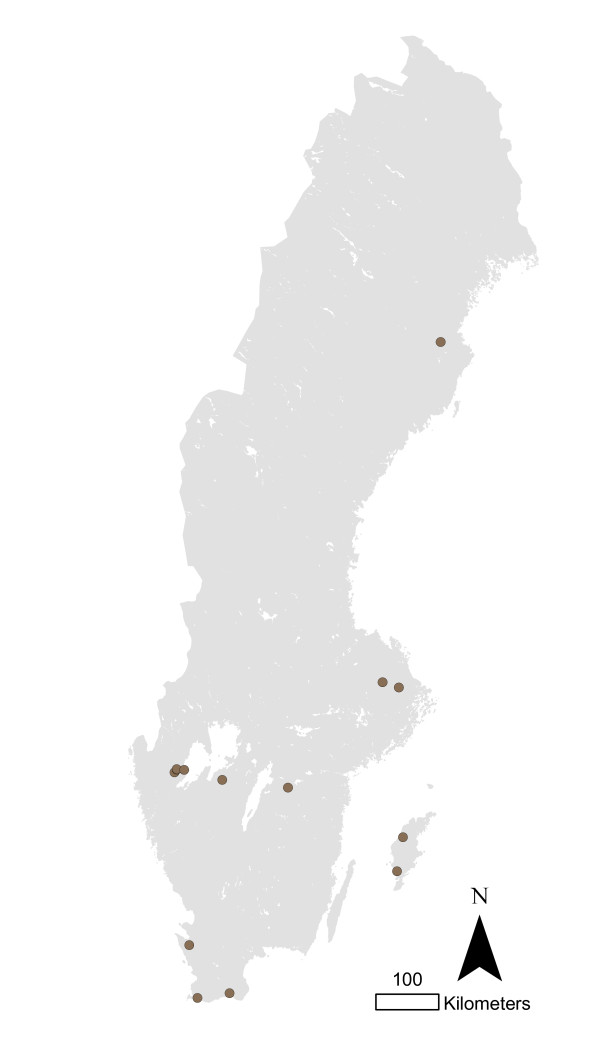
**Pig herds infected with *Salmonella *Typhimurium phage type 40 in Sweden 1993-2010**.

The total numbers of infected herds identified during 1993-2010 in different animal species are shown in figure 1. The most common serotypes identified in different animal species are shown in table 1. The salmonella cases in pigs seemed to be geographically associated with the density of pigs (Figure 2 and 3). The cases of *Salmonella *Typhimurium phage type 40 are shown in figure 4. This serotype has been associated with birds in Northern Europe [7,8] and a geographical clustering would not be unexpected. However, no obvious clustering was found, even when looking at the cases for each year.

**Table 1:** Salmonella serotypes

Animal species	Most common serotype	**2**^**nd **^**most common serotype**	Other serotypes isolated
Cattle	S Dublin (124)	S Typhimurium (45)	Agona, Diarizonae, Dusseldorf, Enteritidis, Jangvani, Livingstone, Montevideo, Mbandaka, Oritamerin, Reading, Sandiego, Stanleyville, Tennessee, Teshie

Pigs	S Typhimurium (31)	S Infantis (6) S Derby (5)	Yoruba, Putten, Newport, Mbandaka, Muenster, Java, Cubana*; Arizonae, Anatum, Agona

Sheep	S Diarizonae (10)	S Typhimurium (2)	Reading

The most common salmonella serotypes isolated from different animals species in Sweden 1993-2010.

*Except for the large outbreak in 2003, S Cubana was only isolated in one pig herd in 1997

**Figure 5:**
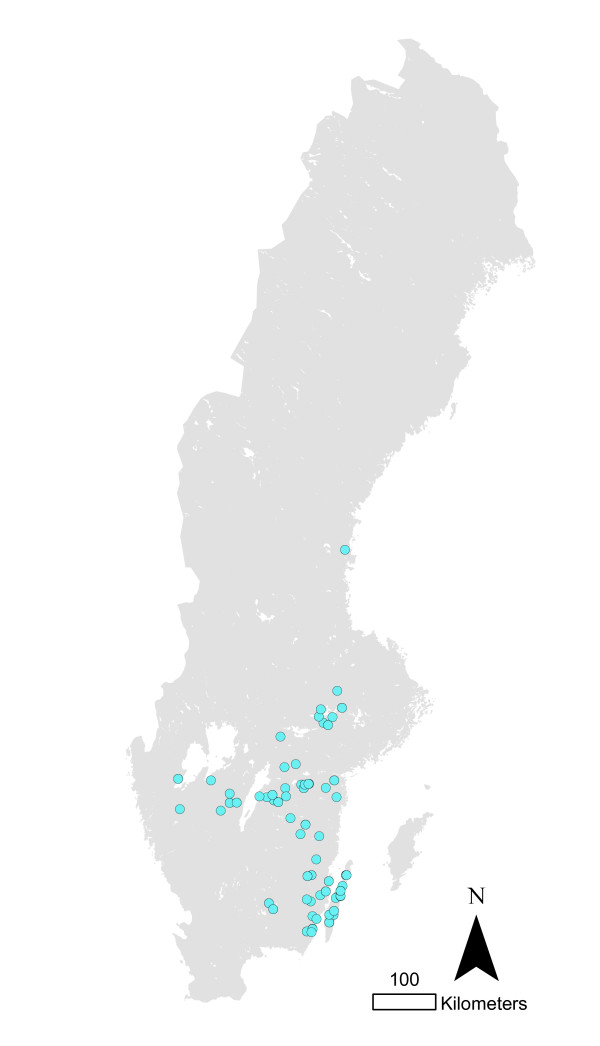
**Cattle herds infected with *Salmonella *Dublin in Sweden 1993-2010**.

**Figure 6:**
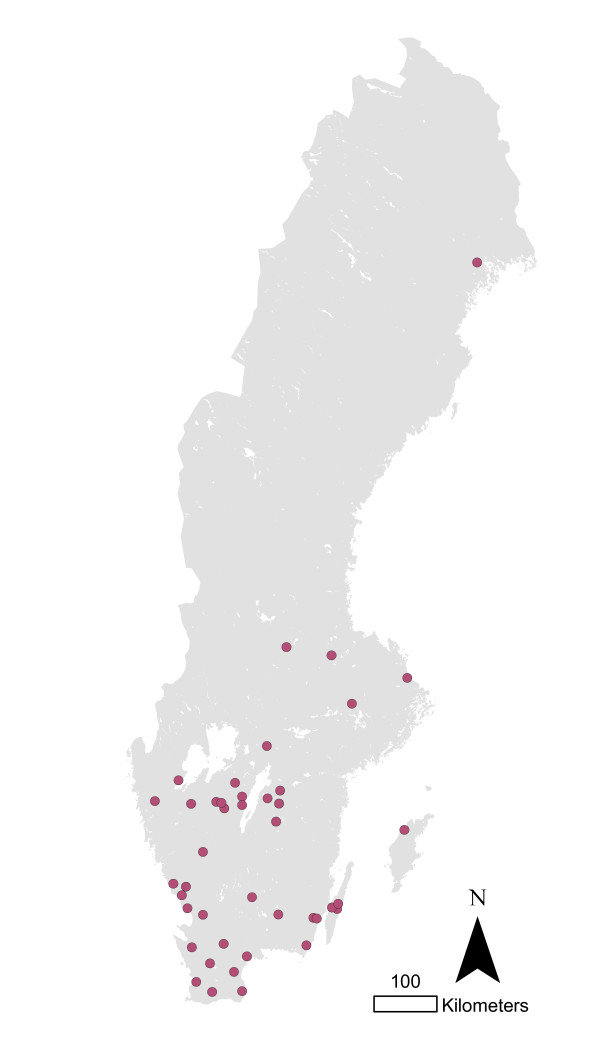
**Cattle herds infected with *Salmonella *Typhimurium in Sweden 1993-2010**.

**Figure 7:**
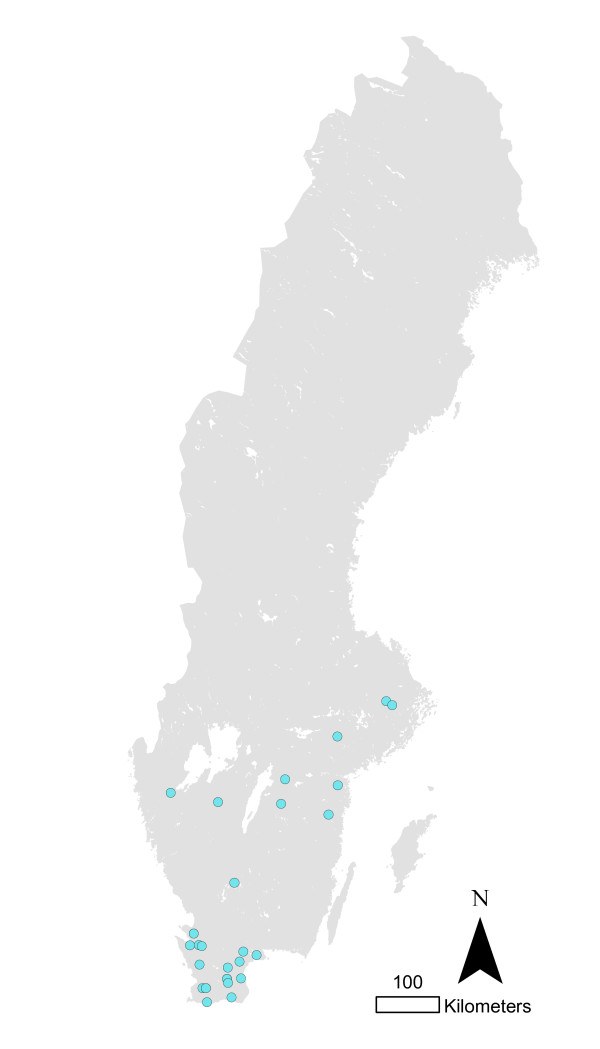
**Cattle herds infected with serotypes other than *Salmonella *Dublin or *Salmonella *Typhimurium in Sweden 1993-2010**.

**Figure 8:**
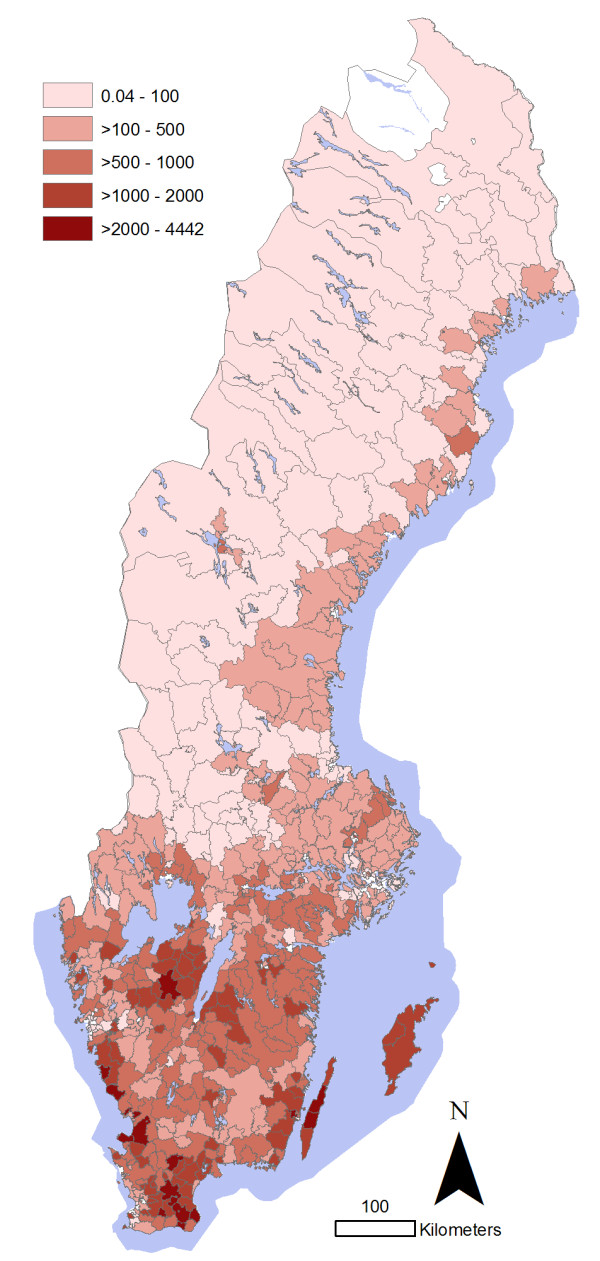
**Density of cattle (i.e. number of animals > 1 year of age per 100 km**^**2**^**) by 3 digit postal code area in Sweden in 2008**. ^© ^Lantmäteriet Gävle 2009, permission number I 2009/0830.

The cases of *Salmonella *Dublin in cattle were located mainly in the southeast region of Sweden (Figure 5), while the majority of the *Salmonella *Typhimurium cases were in the very south (Figure 6) and the other serotypes were more evenly distributed (Figure 7). The overall clustering matched the cattle density (Figure 8).

**Figure 9:**
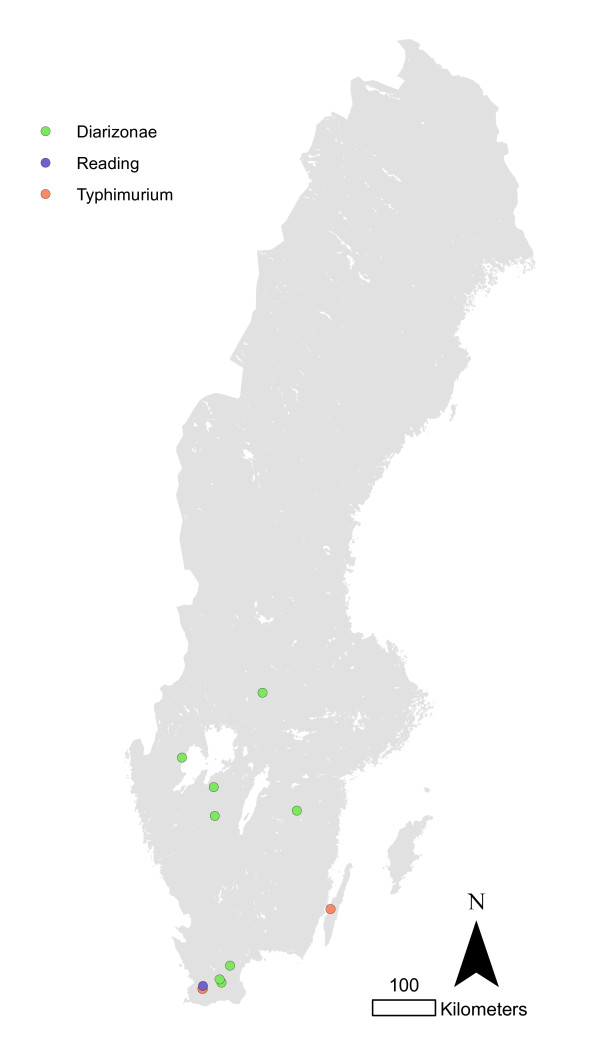
**Salmonella infected sheep herds in Sweden 1993-2010**.

The cases in sheep herds were too few to observe any geographical clustering (Figure 9).

**Figure 10:**
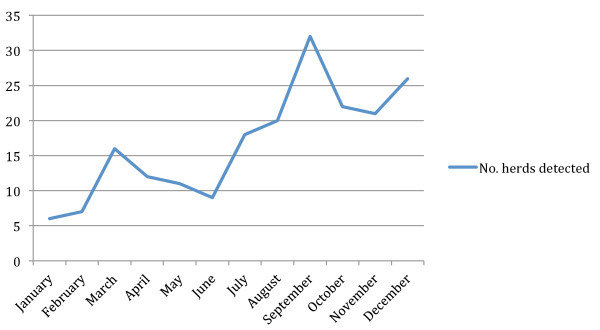
**Number of salmonella infected Swedish cattle herds detected per calendar month**.

Seasonal variation was not investigated for sheep due to the low number of infected herds, and there was neither indication nor reason to suspect seasonal variation in pigs. Some seasonal variation was seen in cattle (Figure 10), but available data was not sufficient for further analyses.

## Discussion

The species where Salmonella was first identified determined the species assigned to the herd. This may not be the same as the species that was first infected and cattle herds may be overrepresented. Complete data on how many herds that had more than one animal species infected was however not available.

By establishing a geodatabase for all case data it has been possible to generate map series from almost any point of view. This facilitated the analysis. The data and the map documents generated can easily be published on a web map server giving access for others to share the results, but also for alternative analysis. The software allows for spatial statistical analysis of the gathered data. In this case, visual inspection of the generated maps proved to be enough to make the conclusions above. How GIS can add new dimensions to epidemiological work has clearly been demonstrated in other studies [11,12].

As the salmonella prevalence in Sweden is low, spatial and temporal analyses of data on infected herds might reveal insights not detectable in endemic regions. However, the low prevalence situation meant that full spatio-temporal analyses were not useful due to the number of herds being too low. Ocular inspection of the resulting maps and diagrams still indicate the presence of some spatial and temporal patterns. For instance, the detection of salmonella infected cattle herds appears to peak in September. The majority of cattle herds are detected through tracing form other infected herds, at necropsy or due to clinical symptoms [13]. It could be speculated that these activities are not as intensive during the summer months due to holidays and harvesting and that the September peak is a reflection of poor detection in the preceding months. However, the increasing number of detected herds in July-August as compared to previous months contradicts this theory. On the other hand, the peak also coincides with some movements of cattle from pasture and may reflect on-farm mixing of animals form various pastures and consequent within-herd spread of infection from a single group of animals exposed at pasture (e.g. via water). There is a seasonal variation in cattle movements in Sweden [14] but the observed pattern of detection of salmonella infected herds does not match this variation, as the peak of infected herds occurs before the autumn peak of movements and the movement peak in spring is not reflected in the number of detected herds. The factors behind the temporal pattern of salmonella infection in cattle merits further investigation.

The geographical clustering of cattle herds infected with *S*. Dublin or *S*. Typhimurium may be a reflection of the clustering of cattle movements in Sweden [15], where the largest number of movements occur within the same geographical region. These two types of salmonella are the most commonly detected in Swedish cattle [6] and the main route of spread between herds would be expected to be via livestock trade. This is particularly true for *S*. Dublin, which is almost exclusively associated with cattle. The geographical clustering is also more pronounced for *S*. Dublin than for *S*. Typhimurium (figures 5 and 6), that may spread via other routes as well.

## Conclusions

Analyses of data on salmonella infected herds revealed some spatial and temporal patterns for salmonella in cattle. However, despite using 18 years' of data, the number of infected herds was too low for any useful statistical analyses and no patterns could be observed in pigs or sheep.

## Competing interests

Lars Skog is employed by ESRI, the company that produces the GIS software used in the study. He is conducting his PhD studies in the Royal Institute of Technology, Geoinformatics in Stockholm, on the use of GIS. Except for this, the authors have no competing interests.

## Authors' contributions

All authors participated in the design of the study. HW and SSL performed the data collection and data cleaning. LS produced the maps of infected herds and JF produced the population density maps. SSL wrote the manuscript and produced the graphs and table. All authors read and approved the final manuscript.

## References

[B1] Sternberg Lewerin S Wahlström H Häggblom P Szanto E Plym Forshell L The Swedish national salmonella control programme - future challenges Proceedings of International Symposium on Salmonella and Salmonellosis (I3S): 10-12 May 2006; St Malo, France 2006 531 534

[B2] Law on Zoonoses, SFS 2006:811 http://62.95.69.15/cgi-bin/thw?%24{HTML}=sfst_lst&%24{OOHTML}=sfst_dok&%24{SNHTML}=sfst_err&%24{BASE}=SFST&%24{TRIPSHOW}=format%3DTHW&BET=1999%3A658%24 (Zoonoslag)

[B3] Ordinance on Zoonoses, SFS 2006:820 http://62.95.69.15/cgi-bin/thw?%24{HTML}=sfst_lst&%24{OOHTML}=sfst_dok&%24{SNHTML}=sfst_err&%24{BASE}=SFST&%24{TRIPSHOW}=format%3DTHW&BET=1999%3A660%24 (Zoonosförordning)

[B4] Regulation on salmonella eradication in animal herds, SJVFS 2004:2 http://www.jordbruksverket.se/download/18.26424bf71212ecc74b080001416/2004-002.PDF (Statens jordbruksverks föreskrifter om bekämpande av salmonella hos djur)

[B5] Eriksson E Aspan A Comparison of culture, ELISA and PCR techniques for salmonella detection in faecal samples for cattle, pig and poultry BMC Vet Res 2007 3 21 10.1186/1746-6148-3-21 17888169 PMC2110889

[B6] Salmonellaportalen, Statistik http://www.sva.se/sv/Fokusomraden1/Salmonellaportal/Statistik/

[B7] Österberg J Vågsholm I Sternberg S Feed-borne outbreak of *Salmonella *Cubana in Swedish pig herds: Risk factors for herds being infected and factors affecting the restriction period in infected farms Acta Vet Scand 2006 47 13 22 10.1186/1751-0147-47-13 16722302 PMC1618960

[B8] Refsum T Handeland K Baggesen DL Holstad G Kapperud G Salmonellae in avian wildlife in Norway from 1969 to 2000 Appl Environ Microbiol 2002 68 5595 5599 10.1128/AEM.68.11.5595-5599.2002 12406754 PMC129881

[B9] Pennycott TW Mather HA Bennett G Foster G Salmonellosis in garden birds in Scotland, 1995 to 2008: geographic region, *Salmonella enterica *phage type and bird species Vet Rec 2010 166 419 21 10.1136/vr.b4761 20364008

[B10] Frössling J Ågren E Eliasson-Selling L Sternberg Lewerin S Probability of freedom from disease after the first detection and eradication of PRRS in Sweden: Scenario-tree modelling of the surveillance system Prev Vet Med 2009 91 137 145 10.1016/j.prevetmed.2009.05.012 19520445

[B11] Skog L Hauska H Linde A The Russian influenza in Sweden in 1889-90: an example of Geographic Information System analysis Euro Surveill 2008 13 pii = 19056 19081003

[B12] Robinson A Assesing Geovisualisation in Epidemiology M.S. Thesis 2005 Pennsylvania State University, Department of Geography

[B13] Lahti E Wahlström H Szanto E Detection of cattle, swine and sheep herds infected with salmonella by different surveillance systems in Sweden during 1993-2009 Proceedings of International Symposium on Salmonella and Salmonellosis (I3S): 28-30 June 2010; St Malo, France 2010 439 440

[B14] Nöremark M Håkansson N Lindström T Wennergren U Sternberg Lewerin S Spatial and temporal investigations of reported movements, births and deaths of cattle and pigs in Sweden Acta Vet Scand 2009 51 37 10.1186/1751-0147-51-37 19811628 PMC2764703

[B15] Widgren S Frössling J Spatio-temporal evaluation of cattle trade in Sweden: description of a grid-network visualization technique Geospatial Health 2010 5 119 130 21080326 10.4081/gh.2010.192

